# High Viral Fitness during Acute HIV-1 Infection

**DOI:** 10.1371/journal.pone.0012631

**Published:** 2010-09-09

**Authors:** Alicia Arnott, Darren Jardine, Kim Wilson, Paul R. Gorry, Kate Merlin, Patricia Grey, Matthew G. Law, Elizabeth M. Dax, Anthony D. Kelleher, Don E. Smith, Dale A. McPhee, and the Pulse Study Team

**Affiliations:** 1 National Serology Reference Laboratory, St Vincent’s Institute, Melbourne, Victoria, Australia; 2 Department of Microbiology, Monash University, Melbourne, Victoria, Australia; 3 Burnet Institute, Melbourne, Victoria, Australia; 4 Department of Microbiology and Immunology, University of Melbourne, Parkville, Victoria, Australia; 5 Department of Medicine, Monash University, Melbourne, Victoria, Australia; 6 National Centre in HIV Epidemiology and Clinical Research, University of New South Wales, Sydney, New South Wales, Australia; 7 St. Vincent’s Centre for Applied Medical Research, Sydney, New South Wales, Australia; University of Sao Paulo, Brazil

## Abstract

Several clinical studies have shown that, relative to disease progression, HIV-1 isolates that are less fit are also less pathogenic. The aim of the present study was to investigate the relationship between viral fitness and control of viral load (VL) in acute and early HIV-1 infection. Samples were obtained from subjects participating in two clinical studies. In the PULSE study, antiretroviral therapy (ART) was initiated before, or no later than six months following seroconversion. Subjects then underwent multiple structured treatment interruptions (STIs). The PHAEDRA study enrolled and monitored a cohort of individuals with documented evidence of primary infection. The subset chosen were individuals identified no later than 12 months following seroconversion to HIV-1, who were not receiving ART. The relative fitness of primary isolates obtained from study participants was investigated *ex vivo*. Viral DNA production was quantified using a novel real time PCR assay. Following intermittent ART, the fitness of isolates obtained from 5 of 6 PULSE subjects decreased over time. In contrast, in the absence of ART the fitness of paired isolates obtained from 7 of 9 PHAEDRA subjects increased over time. However, viral fitness did not correlate with plasma VL. Most unexpected was the high relative fitness of isolates obtained at Baseline from PULSE subjects, before initiating ART. It is widely thought that the fitness of strains present during the acute phase is low relative to strains present during chronic HIV-1 infection, due to the bottleneck imposed upon transmission. The results of this study provide evidence that the relative fitness of strains present during acute HIV-1 infection may be higher than previously thought. Furthermore, that viral fitness may represent an important clinical parameter to be considered when deciding whether to initiate ART during early HIV-1 infection.

## Introduction

HIV-1 exists within the host as a swarm of genetically related strains, termed quasispecies [1]. The heterogeneity of the quasispecies occurs largely as a result of the highly erroneous reverse transcription process [2]. Combined with the rapid rate of virion production (between 10^8^ and 10^9^ virions per day) and the large number of infected cells (10^7^ to 10^8^), the result is a highly diverse HIV-1 population [3], [4], [5]. Additionally, recombination between distinct strains within a host can also occur, further increasing diversity within the virus population [1], [6].

The inherent genetic diversity of HIV-1 facilitates rapid evolution and adaptation to a given or changing environment within the infected host, referred to as viral fitness [6], [7]. Adaptation of HIV-1 involves migration and dissemination throughout the host, escape from adaptive and innate immune responses, and from antiretroviral drug pressure [6]. Fitness therefore is dependent upon viral and host factors, and has been associated with HIV-1 disease progression in individuals with chronic HIV-1 infection [6], [8], [9]. It is thought that individuals harbouring virus isolates that are attenuated or replicate poorly are able to control virus replication and delay disease progression compared with individuals infected with rapidly replicating virus isolates. A correlation between poor *ex vivo* replication and VL suppression was observed following analysis of individuals infected with a *nef*/LTR attenuated strain [9], [10], [11], [12], [13], [14]. In the findings of Trkola et al. (2003), viral fitness of isolates obtained prior to initiation of ART strongly correlated with the degree of VL rebound following treatment cessation in a group of 20 individuals with chronic HIV-1 infection [8]. A strong correlation between *ex vivo* viral fitness and disease progression was demonstrated following analysis of virus isolates obtained from three well characterised long term survivors (LTS) of HIV-1 infection, and three individuals with chronic, progressive HIV-1 infection [15]. Similarly, Campbell et al. (2003) reported a strong linear relationship between HIV-1 replication *ex vivo* and plasma VL for 12 individuals with chronic HIV-1 infection [16]. Collectively, these observations suggest a correlation between *ex vivo* viral fitness and clinical outcome in chronic HIV-1 disease [17].

Little is known regarding viral fitness during the acute phase of infection. From what is known, the fitness of isolates present during acute HIV-1 infection is thought to be low relative to isolates present at later stages of infection, due to the significant genetic bottleneck imposed upon transmission [1], [18]. Indeed, findings from two studies investigating founder viruses and viral diversification in acute HIV-1 infection revealed that in the majority of individuals investigated, infection occurred as a result of transmission or expansion of a single founder virus [19], [20]. The genetic properties required for efficient transmission may differ from those required for effective establishment and dissemination of HIV-1 infection throughout the new host. As a result, the adaptive potential of transmitted strains may be reduced [1].

To examine the relationship between *ex vivo* viral fitness and control of VL in the acute or early chronic stage of HIV-1 infection in this study, viral strains obtained from participants of two clinical cohorts were investigated [21], [22]. Relative viral fitness was assessed using a highly sensitive, quantitative real time PCR (QPCR) assay to measure production of total HIV-1 DNA. Total HIV-1 DNA production can be detected as early as 3 h post-infection *ex vivo*, preceding production of integrated and circular forms [23]. Hence, total HIV-1 DNA production was thought to represent a sensitive, early and reliable marker to assess the relative viral fitness of isolates investigated in this study. We found that *ex vivo* viral replicative fitness did not correlate with coincident plasma VL from individuals in the acute and early chronic stages of HIV-1 infection. Surprisingly, the fitness of isolates obtained from individuals prior to, or immediately following seroconversion to HIV-1 was equal to or greater than that of isolates obtained from ART naïve individuals with early, chronic HIV-1 infection. The results of this study suggest that despite the genetic bottleneck occurring upon transmission of HIV-1, the replication capacity of transmitted strains is not necessarily reduced. As viral pathogenicity has been linked to fitness, the findings of this study also suggest that the pathogenicity of isolates present during acute HIV-1 infection may be higher than previously thought, perhaps providing further evidence for the initiation of ART during this phase of HIV-1 infection.

## Methods

### Patients

Plasma samples were obtained from 20 of 60 participants of the PULSE study [21] (Table S1). The PULSE study was designed to investigate whether individuals with acute HIV-1 infection could suppress HIV-1 replication following multiple structured interruptions (STIs) to ART. Briefly, the PULSE study consisted of four phases: A, B, C and D. Baseline plasma samples were collected from subjects upon enrolment into the study, prior to initiation of ART (Phase A). Subjects received ART [stavudine, lamivudine, ritonavir-boosted indinavir with randomisation to hydroxyurea (HU) or not] until plasma VL decreased to <50 RNA copies/ml for three consecutive months. Patients selected were stratified to ensure a balance of acute or early primary HIV-1 infection (PHI) with or without HU [21]. Once VL was contained below detection in Phase A, subjects underwent carefully monitored STI in Phase B. Subjects remained off ART if the VL remained below 5 000 RNA copies/ml. Once the VL increased above 5000 RNA copies/ml, ART was reinitiated as Phase C. Treatment interruption (Phase B) followed by reinitiation of ART (Phase C), occurred a maximum of three times for each subject, prior to entry into Phase D. Phase D was a follow-up phase, a period of clinical monitoring following the completion of the mandated treatment interruptions study [21].

Seventeen participants of the PHAEDRA study were investigated in parallel with PULSE study subjects (Table S2). The PHAEDRA study was a natural history cohort study, patients could elect to be treated or not. It was established to monitor immunological and virological characteristics of individuals with acute and early HIV-1 infection. Documentation of acquiring HIV within the past 12 months was the criteria for entry. This particular substudy was restricted to a cohort of patients who had elected not to receive ART. All these participants had seroconverted to HIV-1 at enrollment. Samples were collected at baseline and 24, 36 and 52 weeks subsequently. Seroconversion for both cohorts was defined according to stages described by Fiebig and collegues [24] (Tables S1 and S2). For the subjects from whom virus was successfully isolated and further study performed, at baseline the PULSE subjects had a median Fiebig stage of 4 with a mean VL and CD4 T cell count of 1 383 342 RNA copies/mL and 533.5 cells/µl, respectively. At baseline, the median Fiebig stage was 6 for the PHAEDRA subjects, with a mean VL and CD4 cell count of 159 286 RNA copies/mL and 720.7 cells/µl, respectively (Tables S1 and S2).

Plasma samples were stored at −80°C, and patient PBMCs in liquid nitrogen, until required. Research ethics approval was given by St Vincent's Hospital, Sydney, St Vincent's Health, Melbourne and the University of New South Wales Research Ethics Committees. All participants signed an informed consent form before study entry.

### Cells

Peripheral blood mononuclear cells (PBMCs) were isolated by density gradient centrifugation from buffy packs collected from healthy, HIV-1 seronegative individuals, obtained from the Australian Red Cross Blood Service (ARCBS, Melbourne, Australia), as described [25]. Cells were maintained in RF-10 medium (RPMI-1640 medium supplemented with 10% [v/v] heat-inactivated foetal bovine serum, 0.03 µg/ml L–glutamine, 100 U/ml penicillin and 100 µg/ml streptomycin), and activated with 10 µg/ml of phytohemagglutinin (PHA) for 3 days prior to infection with primary HIV-1 strains.

Replication of primary isolates can vary considerably in PBMCs from different donors [26]. To minimise the impact of donor variability, all donor PBMCs used for virus isolation and viral fitness experiments were screened against a diverse panel of primary HIV-1 isolates to determine permissiveness to infection with HIV-1, prior to use. Cells were selected for use in the fitness assay based on the ability to support replication of a genetically diverse panel of primary HIV-1 isolates [9]. The level of CD4 expression on the surface of PBMCs capable of supporting replication of genetically diverse primary HIV-1 strains was significantly higher than on PBMCs that could not (Pate and McPhee, unpublished). To further minimise the effects of donor variability, pooled preferred PHA-PBMCs from two separate HIV-1 negative donors were used for all experiments.

### Viruses

The reference isolate HIV-1_MBC925_ was isolated from PBMCs collected from an AIDS patient, and characterised as described [27]. This highly pathogenic, clade B, CCR5-using primary isolate was selected as it had been observed to replicate efficiently and reproducibly in PHA-PBMCs (McPhee, D.A., unpublished) [27]. The use of HIV-1_MBC925_ also enabled a direct comparison between the fitness of isolates present during acute infection relative with that of an isolate obtained from an individual with advanced disease. Virus isolation was attempted from 36 and 34 plasma samples collected from PULSE and PHAEDRA subjects, respectively, by centrifugation over a 20% (w/v) sucrose cushion at 45 000× *g* for 1 h. Plasma was preferred as it best represents the circulating quasispecies. The pelleted virus was resuspended in IL-2 medium (RF-10 medium containing 10 U/ml IL-2 and 12 mM HEPES) containing 1×10^7^ PHA-PBMCs and cultured for 14 days [25]. Virus production was analysed by measurement of cell-free reverse transcriptase (RT) activity or p24 antigen production.

Virus isolation was attempted from plasma collected at Baseline prior to the initiation of ART, from all PULSE subjects, and from any additional, available Phase B (STI) plasma sample with a VL ≥5 000 RNA copies/ml. Coincident plasma VL measurements ranged from 260 to 7 500 000 RNA copies/ml (Table S1). Isolates were successfully obtained from 19 of the 36 plasma samples: 15 from Baseline and 4 from plasma collected subsequent to Baseline. A strong correlation between plasma virus isolation from PULSE subjects, and high coincident VL, was observed. Virus isolation was unsuccessful from plasma samples with a VL <153 000 RNA copies/ml (Table S1).

Virus isolation was attempted from two plasma samples obtained from each PHAEDRA subject: a sample collected at Baseline and a sample collected at either week 24, 36 or 52 subsequent to Baseline. Two sequential isolates were successfully obtained from 12 of the 17 PHAEDRA subjects (Table S2). Only one isolate, obtained from plasma collected at Baseline, was obtained from an additional PHAEDRA subject (data not shown). Successful virus isolation from plasma obtained from PHAEDRA subjects did not correlate with plasma VL (Table S2). A total of 25 viruses were isolated from PHAEDRA cohort members (Table S2). The relative fitness of 18 of the 25 isolates was subsequently investigated in this study.

Virus isolation was attempted from cryopreserved PBMCs available from a subset of PULSE subjects, where plasma was unavailable, or when virus isolation from plasma was unsuccessful, using co-culture with preferred PBMCs as described above [9]. After recovery from storage in liquid nitrogen, the viability of all PBMCs collected from PULSE subjects and subsequently used for co-culture was ≥70% (data not shown). PBMCs were available from two post-Baseline time-points from six of 10 PULSE subjects, and one post-Baseline time point from a further four subjects, a total of 16 samples. Following co-culture, eight additional isolates were successfully obtained from eight PULSE subjects. There was no correlation between VL and successful virus isolation from PBMCs (Table S1). A total of 28 isolates were obtained from 16 of 20 PULSE subjects; the relative fitness of 24 of the 28 isolates was subsequently investigated.

### Parallel infection assays

A standardised input of 600 pg of p24 antigen of each primary or reference isolate was incubated with 2×10^5^ PHA-PBMCs for 2 h, in triplicate. Isolates were minimally passaged in an attempt to ensure isolates reflected the replication competent virus present *in vivo* [28], [29]. Where 600 pg of p24 could not be achieved, undiluted infection supernatant was added. Cells were washed in IL-2 medium and transferred to 96 well plates at 2×10^5^ cells/well in a final volume of 200 µl, achieved using IL-2 medium. Cells were harvested at various time-points between 0 and 158 h post-infection. Following harvest, cells were washed and resuspended in 200 µl of TE buffer. To lyse infected cells, 300 µl of MagNA Pure lysis buffer (Roche, Castle Hills, NSW, Australia) was added and the cells incubated (15 minutes, room temperature). Lysed cells were stored at −80°C until required for DNA extraction. Harvested supernatant was stored at −20°C and virus production analysed by measurement of cell-free RT activity or p24 antigen production. DNA was extracted from HIV-1 infected PHA-PBMCs using the Invitrogen Easy DNA kit as per the manufacturer's instructions, with the exception of the initial cell lysis step.

### Measurement of total HIV-1 DNA production to estimate relative viral fitness

**Figure 1:**
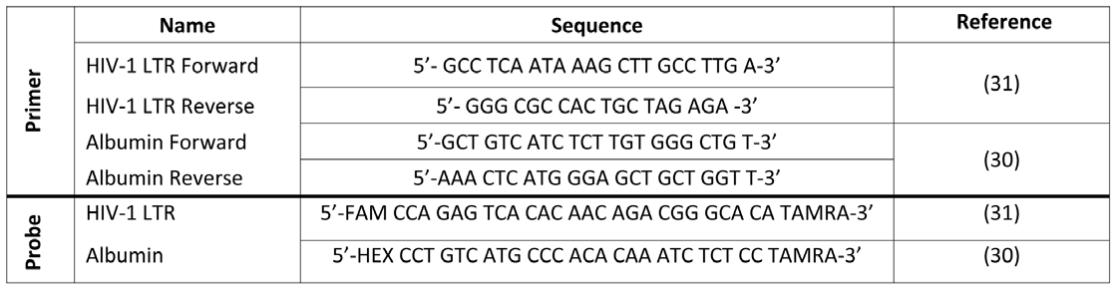
Primers and probes used in this study. The original sources and sequences of the primers and probes used in this study are summarised. The fluorophores used were FAM and HEX for the HIV-1 LTR and albumin probes, respectively. The quencher used for both probes was TAMRA.

To evaluate relative viral fitness, a quantitative real time PCR (QPCR) assay was developed to measure production of total HIV-1 DNA (extrachromosomal, integrated and 2-LTR circular forms) for a period of between 96 and 158 h post-infection. Published primer and probe sequences targeting a highly conserved region of the 5′-LTR and the human Albumin gene, were used (Figure 1) [30], [31]. Prior to use assay sensitivity and intra- and inter-assay variation were extensively tested (Text S1; Tables S3 and S4). To detect total HIV-1 DNA, the real time PCR reaction mix contained 5 µl DNA in a final volume of 20 µl. The mix contained QPCR Probe Mastermix (Integrated Sciences, Australia), 100 nM dual labelled probe, 300 nM of HIV-1 LTR forward and reverse primers, and nuclease free water (NFW). To detect Albumin DNA, the real time PCR reaction mix contained 5 µl DNA in a final volume of 20 µl. The reaction mix contained QPCR Probe Mastermix, 100 nM dual labelled probe, 300 nM Albumin forward and reverse primers and NFW. All DNA amplifications were performed using a Stratagene MX3000P real time PCR machine (Integrated Sciences, Australia) with the following conditions: 1 cycle at 95°C for 10 mins; 40 cycles at 95°C for 30 sec and 60°C for 1 min.

### Quantification and calculation of relative viral fitness scores

As each strain was tested in triplicate, the mean Ct for each time point was calculated following QPCR analysis. Copies of target DNA were quantified by converting the mean Ct value generated for each sample to DNA copies using the standard curve generated by MX3000P software from the quantified DNA standards included in each run. Copies of total HIV-1 cDNA were calculated per 200 000 cells, the number of PHA-PBMCs in each sample. Using the copies of HIV-1 DNA measured for each strain, a relative viral fitness score was calculated for each isolate. Total HIV-1 DNA production at 96 h post-infection was measured for all isolates tested; hence calculation of viral fitness scores at this time-point enabled direct comparison between the relative fitness of all PULSE and PHAEDRA isolates tested. A second score at the final time-point tested (either 110 or 158 h post-infection) enabled analysis of DNA production for those isolates not detected at 96 h post-infection. As the viral fitness measured in this study was relative to that of the pathogenic reference strain HIV-1_MBC925_, fitness scores for test strains were calculated relative to HIV-1 DNA production by HIV-1_MBC925_ from coincident time points.

To calculate viral fitness scores, copies of DNA produced by test strains were divided by copies of DNA produced by the reference strain at a coincident time point post-infection [Fitness score  =  (HIV-1 DNA*_T_*/HIV-1 DNA*_R_*)], where HIV-1 DNA*_T_* and HIV-1 DNA*_R_* correspond to copies of HIV-1 DNA produced by the test and reference strains, respectively. Fitness scores throughout the text and figures are represented as a fraction of 1. Isolates with a relative fitness score of ≥0.1 were classified as fit; isolates with a relative viral fitness score of 0.1 to 0.01 were classified as moderately fit; the relative fitness of isolates with a score of <0.01 was classified as low.

## Results

### Reduced relative fitness of a *nef*/LTR attenuated virus compared with a primary wild type HIV-1 strain, HIV-1_MBC925_

**Figure 2:**
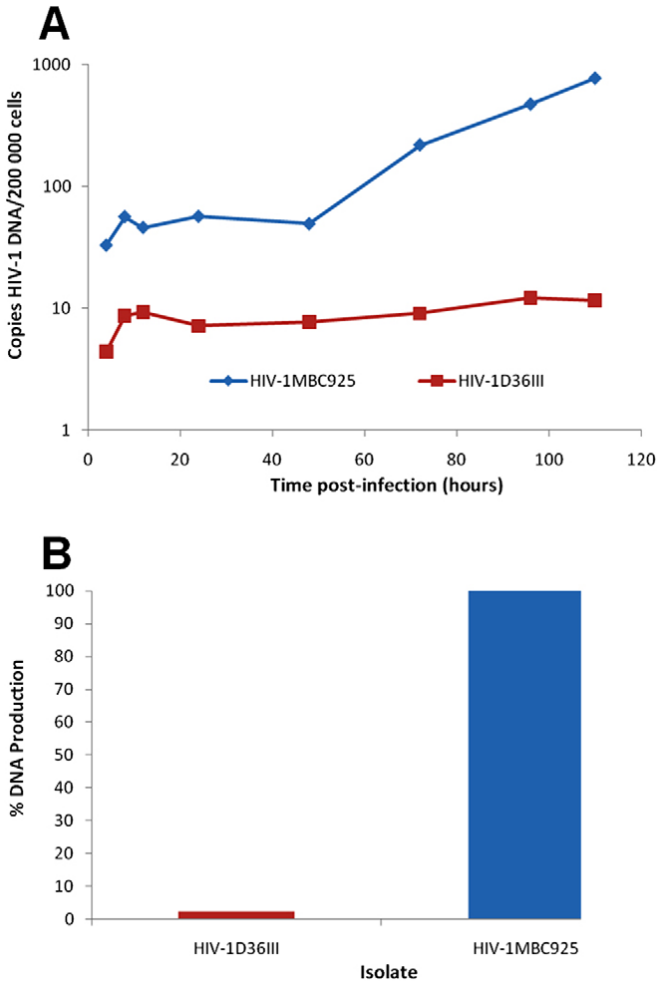
Production of HIV-1 DNA by a reference and a known attenuated virus, quantified using the QPCR assay. PHA-PBMCs were infected with 6 000 pg of p24 of the reference strain HIV-1_MBC925_ and known attenuated isolate HIV-1_D36III_, and cultured for 110 h. Infected cells were harvested at 4, 8, 12, 24, 48, 72, 96 and 110 h post-infection. DNA was extracted and the HIV-1 and albumin DNA quantified using QPCR. Copies of HIV-1 DNA per 200 000 cells, determined for each isolate, are plotted on a logarithmic scale against time (A). In (B), relative fitness of HIV-1_D36III_ was determined by calculating the amount of HIV-1 DNA produced at 96 h post-infection, expressed as a percentage of HIV-1_MBC925_ DNA production at the same time-point. The results are representative of three experiments.

Whether primary HIV-1 isolates of variable replicative fitness could be distinguished on the basis of total HIV-1 DNA production was investigated using HIV-1_MBC925_ and a *nef*/LTR attenuated isolate, HIV-1_D36III_. The HIV-1_D36III_ isolate, obtained from a long term non-progressor (LTNP), replicates poorly, as a result of deletions/mutations in the *nef*/LTR region [9], [11], [32]. Production of viral DNA by both isolates was detected at four h post-infection, however a significant difference in replicative fitness over time was observed (Figure 2). The difference between total HIV-1 DNA produced by HIV-1_MBC925_ and HIV-1_D36III_ at 96 h post-infection was 38.7-fold (Figure 2). It has been observed by studies in our laboratory, and those by Kim and collegues, that a single replication cycle takes between 20 and 24 h [33], [34]. Hence, several rounds of infection were required to demonstrate differences in the kinetics of total HIV-1 DNA production. A slow/low replication phenotype was observed for the attenuated virus strain compared with a fast/rapid DNA production profile for the reference virus. Increased DNA production at all time-points tested by HIV-1_MBC925_ relative to HIV-1_D36III_ indicated that the replicative fitness of the reference strain was greater than that of the attenuated isolate. For both virus infections there was an increase followed by a modest decrease between 4 and 12 h post infection as observed previously in a study of one step growth kinetics of HIV-1 [34]. Furthermore, these results indicated that using the QPCR assay, primary HIV-1 isolates with variable replicative fitness could be readily distinguished on the basis of viral DNA production over several rounds of replication.

### Decreased replicative fitness from acute to early chronic HIV-1 infection, following treatment with ART (PULSE subjects)

**Figure 3:**
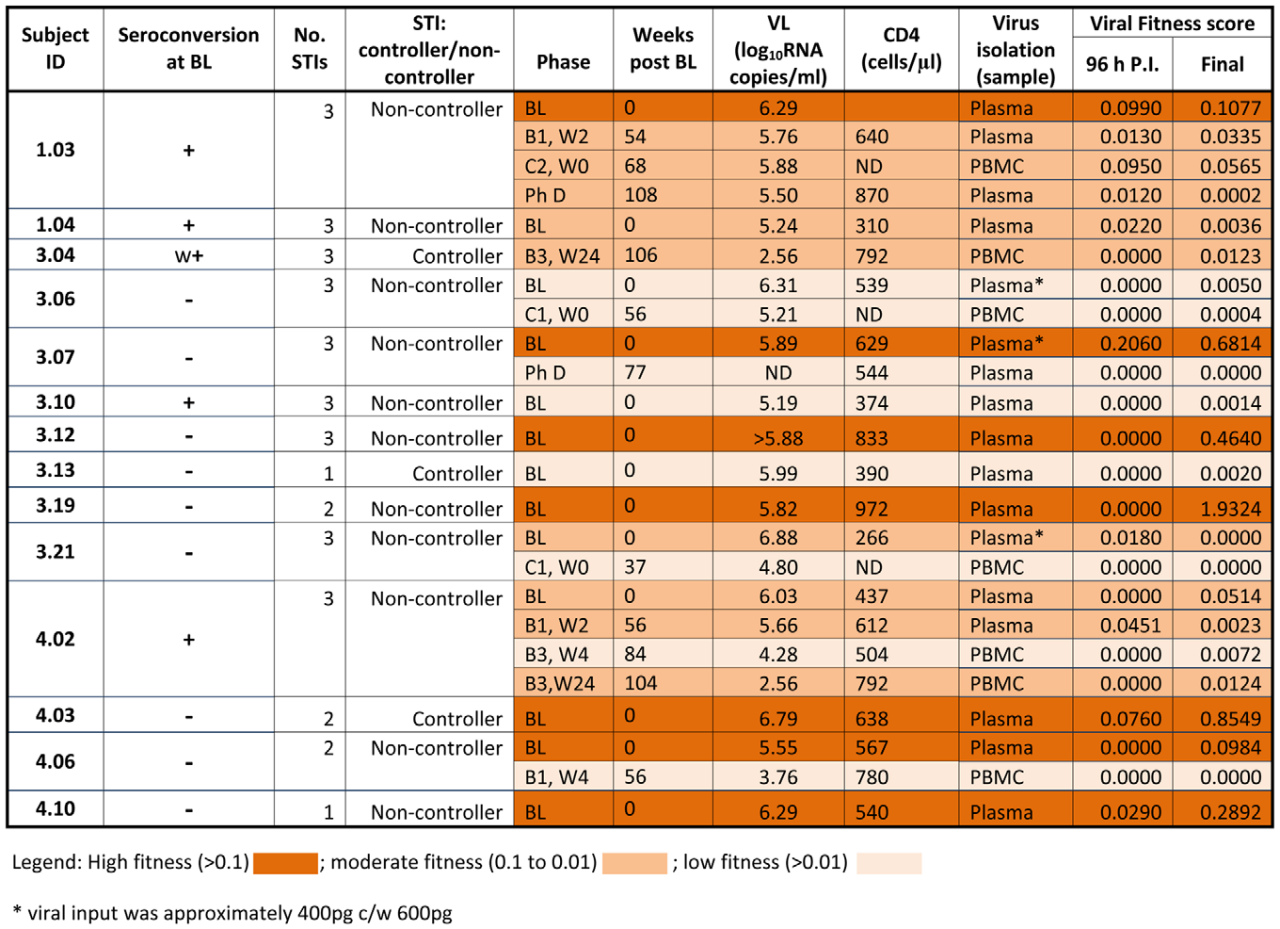
Viral fitness scores and clinical data for PULSE subjects. Shown are clinical and experimental data obtained for PULSE subjects from which virus was successfully isolated and subsequently tested using the real time PCR assay. Indicated by the column headings are the subject identification code and seroconversion status at Baseline (‘+’ indicates subject had seroconverted, ‘−’ indicates subject was seronegative, ‘w+’ indicates that a weak antibody response was detected). Also shown are the number of STIs experienced by the subject, whether VL was suppressed below 5 000 RNA copies/ml upon STI (indicated by ‘controller’ or ‘non-controller’), and the phase of the PULSE study during which the relevant sample was collected. The time (in weeks) post Baseline that the sample was collected, coincident VL and CD4^+^ T cell counts and the sample type from which virus was successfully isolated, are also shown. Finally, viral fitness scores calculated using DNA production measured at 96 h post-infection *ex vivo*, and at the final time-point analysed (158 h post-infection), are shown for each isolate. The fitness scores generated for the isolate obtained from subject 3.13 were calculated from total HIV-1 DNA produced at 60 and 72 h post-infection. ‘ND’ indicates that the specified measurement was not done.

The replicative fitness of isolates obtained from 14 PULSE subjects was investigated using the QPCR assay. The reference isolate HIV-1_MBC925_ was cultured in parallel with test isolates in each viral fitness experiment, enabling calculation of a relative fitness score and to monitor any potential inter assay variation. Two isolates from different time-points obtained from 6 PULSE subjects, and 8 single isolates obtained from 8 PULSE subjects, were tested (Figure 3). From the viral fitness scores calculated using total HIV-1 DNA production at 158 h post-infection, isolates obtained from PULSE subjects were categorised into three groups: high fitness, moderate fitness and low fitness (Figure 3). The seven isolates classified as highly fit were all obtained from plasma collected at Baseline, during acute HIV-1 infection, and prior to initiation of ART. For three BL isolates replication was near equivalent to the reference strain used (Figure 3).

Four of the 8 isolates classified as moderately fit were obtained from plasma collected at Baseline, four were obtained from plasma collected during STI, subsequent to Baseline. The relative fitness of eight isolates was classified as low, indicating that total HIV-1 DNA production by these isolates was less than 1% of total HIV-1 DNA production by the reference isolate at a coincident time-point post-infection *ex vivo* (Figure 3). Six of the 8 isolates with low fitness were obtained from plasma collected during STI. Viral DNA was only detected after 96 h post-infection for these 8 isolates. The rapid kinetics of HIV-1 DNA production after 96 h post-infection lead to selection of 158 h post-infection as the final timepoint for analysis of relative fitness in subsequent experiments (data not shown).

**Figure 4:**
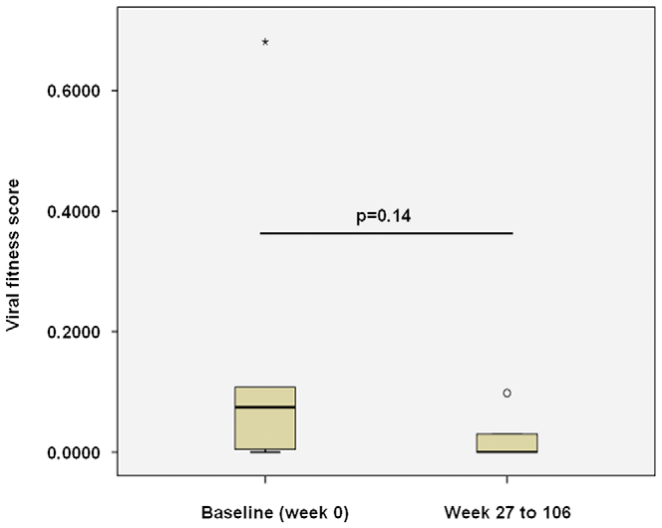
Decreasing fitness over time observed following analysis of paired isolates obtained from acute HIV-1 infection subjects, measured using QPCR. Relative viral fitness scores were calculated for isolates obtained from PULSE subjects and represented on a box-plot. Only subjects from whom a Baseline isolate and at least one additional isolate (Week 27 to 106) were obtained were included in the analysis (n = 6). Where multiple isolates from additional time-points were obtained, the average of the combined viral fitness scores was used. Shown are viral fitness scores calculated at the final time-point tested (158 h; exception was 3.13 which was at 72 h) *ex vivo* for paired isolates obtained from 6 PULSE subjects. The box represents the middle 50% of values for the data set, the solid line indicates the median value. The vertical ‘whiskers’ extending from the box respectively indicate the lowest and highest observed values. The open circle represents an outlier; the asterisk represents an extreme outlier. The significance of the observed changes in viral fitness over time is shown (p = 0.14), calculated using a signed rank test.

**Figure 5:**
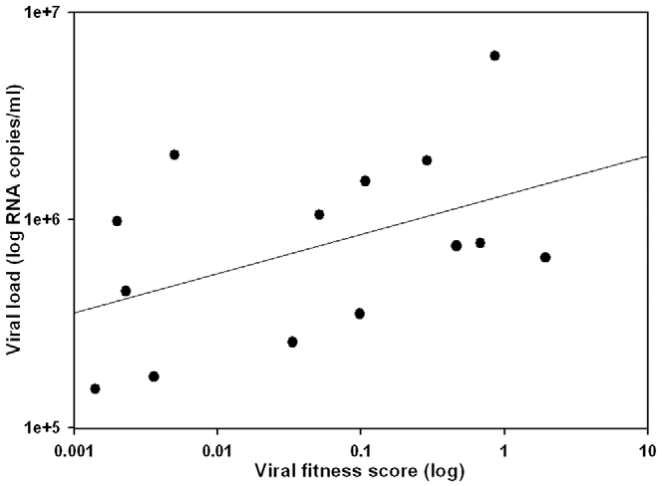
Viral fitness did not correlate with VL following analysis of isolates obtained from acute HIV-1 infection subjects. Coincident plasma VL measurements (log_10_ RNA copies/ml) were plotted against relative viral fitness scores (log_10_) for 16 isolates, obtained from plasma, from PULSE subjects. The Pearson correlation was rho = 0.496, p = 0.051.

Over time, following the initiation of ART, the fitness of isolates obtained from 6 PULSE subjects decreased (Figure 4). Due to the small number of subjects analysed, the decrease observed was not significant (p = 0.14). Furthermore, although decreasing viral fitness coincided with decreasing plasma VL for 4 of 6 subjects from whom multiple isolates were obtained, overall, viral fitness did not correlate with plasma VL for the 14 PULSE subjects investigated (Borderline statistical significance p = 0.051; Figure 5). There was no correlation between CD4+ T cell counts and relative viral fitness for the PULSE subjects investigated (data not shown).

### Increasing replicative fitness during chronic HIV-1 infection (PHAEDRA subjects)

**Figure 6:**
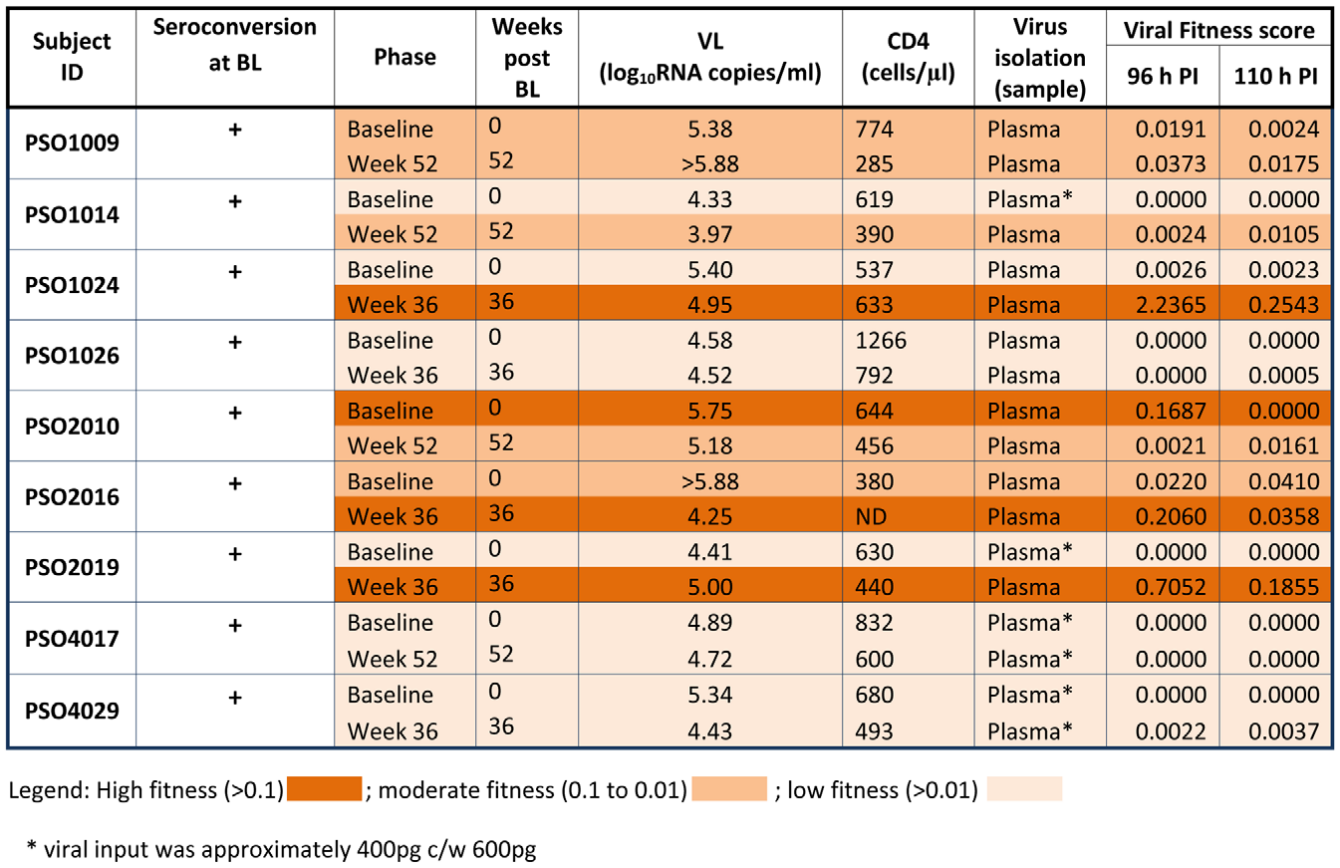
Clinical and experimental data obtained for PHAEDRA subjects. Shown are clinical and experimental data obtained for PHAEDRA subjects from which virus was successfully isolated and subsequently tested using the real time PCR assay. Indicated by the column headings are the subject identification code, seroconversion status at Baseline (‘+’ indicates subject had seroconverted, ‘-’ indicates subject was seronegative), and the phase of the PHAEDRA study at which the relevant sample was collected. The time (in weeks) post Baseline that the sample was collected, coincident VL and CD4^+^ T cell counts, and the sample type from which virus was successfully isolated are shown. The viral fitness scores calculated using DNA production measured at 96 h post-infection *ex vivo*, and at the final time-point analysed (110 h post-infection), are shown for each isolate.

Sequential isolates from 9 of 12 PHAEDRA subjects were analysed using the QPCR assay (Figure 6). Based on relative viral fitness scores calculated using total HIV-1 DNA production at 110 h post-infection, isolates were classified according to the 3 groups used above. Four isolates were categorised as highly fit, 3 of which were obtained from plasma collected 36 weeks subsequent to Baseline (Figure 6). In contrast, all of the isolates obtained from PULSE subjects that were classified as fit were obtained from plasma collected at Baseline, prior to the initiation of ART (Figure 3).

Of the 5 PHAEDRA isolates classified as moderately fit, 3 isolates were obtained from plasma collected 52 weeks subsequent to Baseline, and 2 isolates were obtained from plasma collected at Baseline (Figure 6). The relative fitness of 9 isolates obtained from PHAEDRA subjects was classified as low. Interestingly, 6 of the 9 isolates with low relative fitness were obtained from plasma collected at Baseline, in contrast to results obtained for the PULSE subjects investigated.

**Figure 7:**
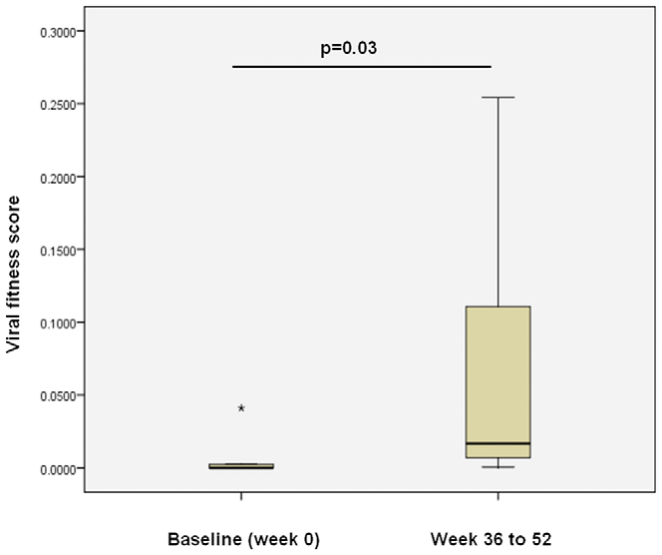
Increasing viral fitness over time observed following analysis of paired isolates obtained from early chronic HIV-1 infection subjects, measured using QPCR. Relative viral fitness scores were calculated for isolates obtained from PHAEDRA subjects and represented on a box-plot. Only subjects from whom a Baseline isolate and at least one additional isolate (Week 36 to 52) were obtained were included in the analysis (n = 8). Shown are viral fitness scores calculated at the final time-point tested (110 h) *ex vivo* for paired isolates obtained from 8 PHAEDRA subjects. The box represents the middle 50% of values for the data set, the solid line indicates the median value. The vertical ‘whiskers’ extending from the box respectively indicate the lowest and highest observed values. The asterisk represents an extreme outlier. The significance of the observed changes in viral fitness over time is shown (p = 0.03), calculated using a signed rank test.

**Figure 8:**
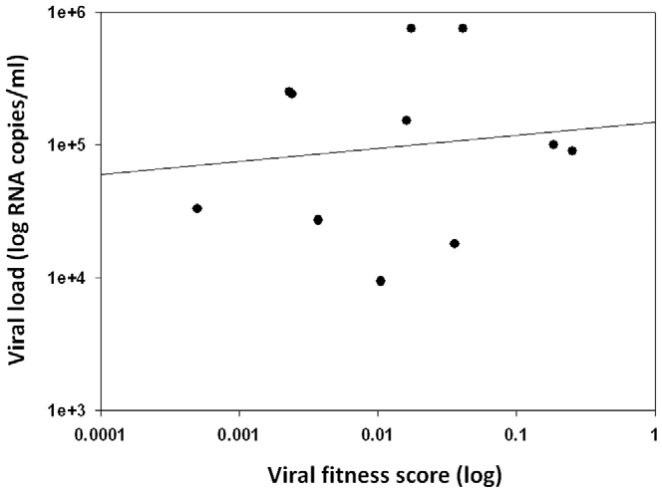
Viral fitness did not correlate with VL following analysis of isolates obtained from early chronic HIV-1 infection subjects. Coincident plasma VL measurements (log_10_ RNA copies/ml) were plotted against relative viral fitness scores (log_10_) for 16 isolates, obtained from plasma, from PHAEDRA subjects. The Pearson correlation was rho = 0.133, p = 0.697.

Over time, the relative fitness of isolates obtained from seven PHAEDRA subjects increased significantly (p = 0.03; Figure 7). However, viral fitness was not found to correlate with plasma VL following analysis of the 18 isolates obtained from PHAEDRA subjects (Figure 8). In addition, relative viral fitness was not found to correlate with CD4+ T cell counts for the PHAEDRA subjects investigated (data not shown).

### High relative fitness of isolates from acute infection (PULSE) compared with early chronic HIV-1 infection (PHAEDRA)

It is widely believed that, due to a genetic bottleneck occurring upon transmission, the fitness of isolates present during acute infection is low relative to isolates obtained later in infection [6], [35], [36], [37], [38], [39]. To investigate this, we compared the viral fitness of isolates obtained from Baseline plasma from PULSE subjects, to those collected 36 to 52 weeks post-baseline from PHAEDRA subjects. The isolate groups were selected to enable the relative fitness of viruses present during acute HIV-1 infection, naïve to any selection pressures exerted by ART (PULSE), and those found during untreated, early chronic infection (PHAEDRA), to be compared.

**Figure 9:**
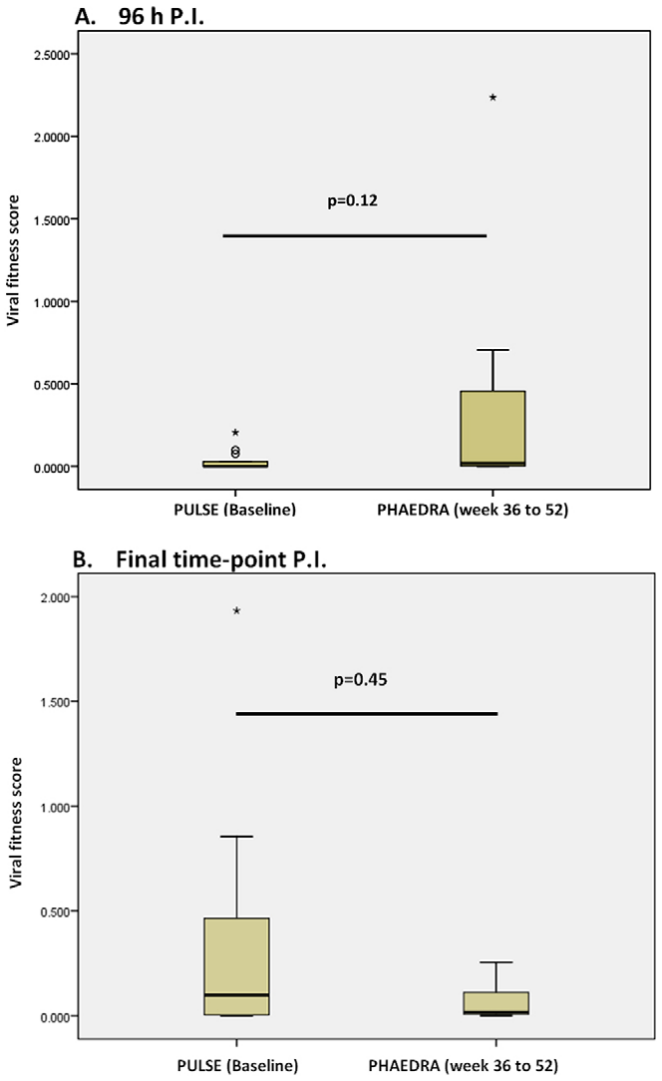
Increased fitness of Baseline isolates obtained from acute HIV-1 infection subjects relative to isolates obtained subsequent to Baseline from early chronic HIV-1 infection subjects. Relative viral fitness scores were calculated for isolates obtained from PULSE and PHAEDRA subjects. Shown on a box-plot are the viral fitness scores generated for the Baseline isolates obtained from 13 PULSE subjects compared with the viral fitness scores of the ‘Late’ isolates obtained from eight PHAEDRA subjects, at 96 h PI (A) and at the final time-point tested (158 h PI for PULSE and 110 h PI for PHAEDRA isolates; B). The box represents the middle 50% of values for the data set; the solid line indicates the median value. The vertical ‘whiskers’ extending from the box respectively indicate the lowest and highest observed values. Outliers are represented by an open circle; extreme outliers are represented by an asterisk. The significance of difference in viral fitness between the two groups at 96 h PI (A; p = 0.12) and the final time-point tested (B; p = 0.45) is shown.

**Figure 10:**
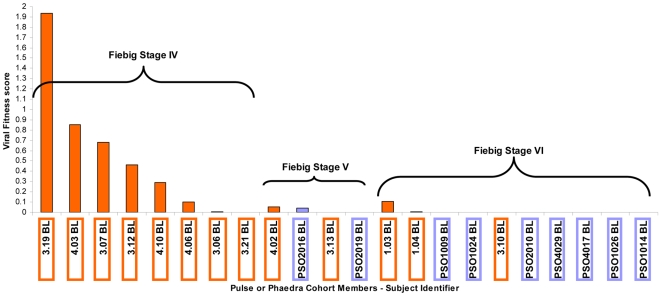
Viral fitness of baseline isolates obtained from acute HIV-1 infection subjects (PULSE) and from early chronic HIV-1 infection subjects (PHAEDRA) relative to the stage of seroconversion as detailed by Fiebig and collegues (24). Baseline viral fitness scores for both PULSE (orange) and PHAEDRA (mauve) subjects from final timepoints (110 h or 158 h) have been grouped according to the stage of seroconversion (Fiebig stages IV, V or VI; reference 24) for direct comparison.

Relative to isolates obtained from PHAEDRA subjects, replication of PULSE Baseline isolates was considerably slower, with replication of 57% of isolates not detected by 96 h post-infection. However, between 96 and 110 or 158 h post-infection, total HIV-1 DNA production increased substantially, with replication of 84% of PULSE viruses detected (Figure 3). By comparison, replication of 39% of PHAEDRA post-baseline isolates was not detected by 110 h post-infection *ex vivo* (Figure 6). In addition, the increase in total HIV-1 DNA production between 96 and 110 h post-infection for isolates obtained from PHAEDRA subjects was not substantive relative to wild type or the isolates obtained from PULSE subjects (data not shown). We observed that overall, PULSE Baseline isolates were slower to establish a productive infection relative to the PHAEDRA post-baseline isolates (Figure 9). From this we suggest that the genetic diversity of isolates obtained post-Baseline from the PHAEDRA subjects was greater than that of the PULSE isolates obtained at Baseline, evidenced by greater relative adaptive ability. However, once infection was established, the amount of HIV-1 DNA produced by the PULSE Baseline isolates was comparable to, or higher than, the amount of viral DNA produced by PHAEDRA post-Baseline isolates (Figure 9). These findings provide evidence that the relative fitness of isolates present during acute HIV-1 infection may be higher than previously thought. When viral fitness scores were plotted relative to the stage of seroconversion the results are even more striking. The most fit viruses were observed during the earliest stage of seroconversion monitored (Figure 10). Conceivably, *in vivo* viral fitness is compromised as HIV-1 infection progresses, in response to selective immunological pressure on replicating virus.

## Discussion

In this study we investigated the relative viral fitness of isolates obtained from individuals with acute and early HIV-1 infection. Temporal changes in relative viral fitness were observed for 6 and 10 subjects participating respectively in the PULSE (acute HIV-1 infection) and PHAEDRA (early HIV-1 infection) studies (Figures 3, 4, 6 and 7). Consistent with the findings of previous studies investigating viral fitness during untreated HIV-1 infection [6], [15], [36], the relative fitness of paired isolates obtained from 7 PHAEDRA subjects increased significantly over time (p = 0.03; Figure 7). Viral fitness decreased over time following intermittent ART for 5 of the 6 PULSE subjects analysed (Figure 4), an observation that might be expected due to the potential bottleneck imposed by suppressive ART. Most unexpected was the high relative fitness of isolates obtained from PULSE subjects during acute HIV-1 infection, prior to the initiation of ART, compared to isolates obtained from individuals with early chronic HIV-1 infection. Furthermore, total HIV-1 DNA production by several PULSE Baseline isolates was comparable to, or greater than that of the highly pathogenic, primary reference isolate HIV-1_MBC925_ obtained from an individual with AIDS (Figure 3) [27]. These findings provide evidence that despite the bottleneck occurring upon transmission, the relative fitness of isolates present during acute HIV-1 infection may indeed be high.

To investigate relative viral fitness, a ‘parallel infection assay’ was used [17]. Parallel infection assays have been successfully used in other studies to examine replication of primary HIV-1 isolates in primary cell types [35], [40], [41]. Alternatively, viral fitness can be investigated using a growth competition assay, whereby replication of test and reference strains is compared in the same culture, primarily performed using recombinant viruses [6], [15], [17], [42], [43], [44], [45]. The use of recombinant strains, as in recent studies by Miura et al., [46] and Kong et al., [47] to investigate the contribution of specific genes to the fitness of viruses during acute infection, does not permit investigation of the fitness of the circulating viral quasispecies. We used a parallel infection assay to enable investigation of the replicative fitness of strains isolated directly from patient plasma, to maximise the clinical relevance of results obtained [8].

It is widely accepted that regardless of the route of HIV-1 infection, the virus encounters an extreme genetic bottleneck upon transmission, resulting in a highly homogenous virus population in the recipient [19], [37], [38], [39], [48]. Decreased genetic diversity is thought to activate Muller's ratchet [49], therefore, the fitness of strains present during acute infection is thought to be low. As 10 of the 20 PULSE individuals investigated had not fully seroconverted to HIV-1 (Table S1), we anticipated that the fitness of viruses isolated from coincident plasma samples would be low. A virus population with highly constrained genetic diversity would not be expected to readily adapt to an environment distinct to that found within the host, such as the *ex vivo* system used in this study to measure relative viral fitness [6].

However, 7 of the 13 isolates obtained from plasma collected at Baseline from PULSE subjects were classified as highly fit (Figures 3 and 9). Indeed, analogous to the findings of this study, rapidly replicating variants have been identified in similar, smaller studies investigating the fitness of isolates present during acute and early HIV-1 infection [6], [36], [40]. In the findings by Ferbas et al. (1996) for one individual, high viral fitness was observed following analysis of the ex vivo fitness of isolates obtained at the time of peak viremia, but prior to seroconversion [36]. Kong et al. (2008) recently reported that strains with higher replicative fitness with respect to the *env* gene were vertically transmitted by mothers with chronic HIV-1 infection [47]. Combined with the observation of highly fit strains present during acute HIV-1 infection in this study, these results suggest the bottleneck that occurs upon initial transmission of HIV-1 does not necessarily result in loss of fitness.

The level of relative viral fitness has been linked to the genetic diversity of the viral quasispecies. Kong et al. (2008) reported transmission of multiple virus strains; Borderia et al. (2010) recently demonstrated a direct correlation between increasing genetic diversity and increasing in vivo viral fitness of clonal populations [47], [50]. Troyer et al. (2005) reported strong correlation between genetic diversity of the viral quasispecies, and ex vivo viral fitness [6]. In our study, with subjects that were therapy naive, viral fitness increased over time for 7 of the 9 PHAEDRA subjects investigated. Observations that genetic diversity correlates with viral fitness are certainly not novel; fitness of an RNA virus population increasing with genetic diversity is described by the Red Queen hypothesis [51]. This has been applied extensively in the field of HIV-1 research [7], [40], and is highly relevant given the level of genetic diversity of the viral quasispecies present in infected individuals. Cloning of the *env* sequences of isolates obtained from PULSE subjects is currently underway, to investigate whether the observed high level of fitness correlated with genetic diversity of the quasispecies present at baseline, during acute infection.

Following commencement and subsequent interruption of suppressive ART, viral fitness decreased for 5 of 6 PULSE subjects investigated (Figure 3). Analogous to the findings of this study, reduced viral fitness was also observed for individuals experiencing STI following initiation of ART during acute infection by Wang et al. (2007) [52]. Suppressive antiretroviral therapy can result in the development of drug resistant mutations in the viral quasispecies to evade inhibition, which has been shown to reduce viral fitness [6], [21]. Development of drug resistance mutations in this study was not suspected as VL suppression was observed upon resumption of ART in all PULSE subjects investigated [21]. Instead, analogous to the findings of Wang et al., (2007) [52] and Borderia et al. (2010) [50], decreasing relative viral fitness over time was thought to be a direct result of a genetic bottleneck created by suppressive ART, activating Muller's ratchet [6]. Muller proposed that when genetically diverse populations are randomly reduced, such as during treatment with ART, or the development of potent immune responses, the overall fitness of the population also decreases [6], [40]. The fitness of Baseline isolates obtained from 6 of the 9 PHAEDRA subjects was also classified as low (Figure 6). At Baseline, all PHAEDRA subjects had clearly seroconverted to HIV-1 (Table S2). The observed low relative fitness may have resulted from mutation of the viral quasispecies as a direct result of the development of potent immune responses following seroconversion. Indeed, escape from targeted immune responses has been observed in similar studies investigating anti-HIV-1 immune responses during early HIV-1 infection [17], [53].

The accumulation of escape mutations can incur a high fitness cost to the virus, depending on the genomic location of the mutation [54], [55], [56]. Indeed, Goonetilleke and colleagues (2009) reported that selection of viral escape mutants, following development of adaptive T-cell responses, occurred rapidly following containment of peak viremia in 4 individuals with acute HIV-1 infection confirming earlier studies [57], [58], [59]. However, there was no obvious fitness cost to the viruses studied [59]. Similarly, as relative viral fitness increased subsequent to Baseline for 7 of 9 PHAEDRA subjects investigated in this study, accumulation of deleterious mutations seems unlikely. Not as restrictive as suppressive ART, development of potent immune responses upon seroconversion may have created a “wider” bottleneck, limiting but not preventing the expansion and diversification of the viral quasispecies [6]. Consequently, we propose that increasing fitness subsequent to seroconversion observed for 7 of 9 PHAEDRA subjects occurred as a result of virus evolution and diversification within the host to evade adaptive immune responses [6], [7], [40], [51]. Although contribution of cellular immune responses to containment of virus replication has not been investigated we are currently assessing neutralising antibody responses for both the PULSE and the PHAEDRA subjects.

There were several limitations to the present study. The use of an *ex vivo* system, such as that used in this and other studies, does not reflect the sensitivity of the virus to antiretroviral drugs, chemokines or additional inhibitory agents that may affect fitness *in vivo*. Furthermore, for 6 of the 14 PULSE subjects from whom plasma virus could not be isolated, virus was isolated from PBMC (Figure 3). In addition to PBMC-derived isolates, for 5 of these 6 subjects, virus was obtained from plasma collected at distinct time-points throughout the study. The fitness of both PBMC and plasma derived viruses was subsequently investigated (Figure 3). It has long been understood that HIV-1 can evolve separately in distinct physiological compartments [60], [61]. In addition, it is a widely held belief that the current, circulating viral quasispecies are present in the plasma and that cellular reservoirs of HIV-1 contain archived strains. However, the findings of recent studies suggest otherwise [62], [63]. Indeed, we observed that the kinetics of HIV-1 DNA production by the PBMC-derived isolates tested in this study were distinct relative to plasma derived isolates obtained at different time-points from the same PULSE subject (data not shown).

Combined, observations of the relative fitness of PULSE and PHAEDRA isolates suggest selection of the fittest virus, or viruses, upon transmission which progressively become less fit upon development of adaptive immune pressure and/or commencement of antiviral therapy. Further studies to investigate the long-term impact of viral fitness on disease progression are warranted. Muira and collegues recently reported the attenuated replication capacity of isolates obtained from individuals who became HIV-1 controllers during early infection [46]. In this study, none of the PULSE subjects from whom Baseline isolates with high replicative fitness were obtained controlled HIV-1 replication in the absence of therapy (data not shown). Although the role of viral fitness in disease progression remains unclear, what is clear from the findings of this study is that the fitness of strains present during acute/early HIV-1 infection can be high.

In conclusion, the findings of this study suggest that despite the bottleneck transmission of a strain or strains with high relative fitness does occur. Furthermore, these results suggest that viral fitness decreases subsequent to the development of adaptive immune pressure and/or commencement of antiviral therapy. The findings of this study make a substantial contribution towards understanding that the selection process during transmission of HIV-1 from donor to recipient can be for a very fit virus.

## Supporting Information

Text S1 Detail of viral fitness QPCR assay validation.
(0.04 MB DOC)

Table S1 Clinical and virus isolation data for PULSE subjects. Shown are the clinical results and the results of attempted virus isolation from plasma or PBMCs obtained from PULSE subjects; “triangle” indicates that virus isolation was attempted from the sample indicated. A single asterisk indicates the sample used for virus isolation was plasma; a double asterisk indicates that virus isolation was attempted from PBMCs when either plasma was not available or virus isolation from plasma was unsuccessful. Shown is the subject identification number followed by the phase of the PULSE study during which the sample was collected. “A”, “B” and “C” indicate PULSE study Phases A, B and C. The subsequent number indicates during which of up to three B or C phases sample collection occurred; prefaced by “W” (weeks), the following number indicates duration of the specified phase at sample collection. Seroconversion status according to the Fiebig et al [24] stages, coincident CD4+ T cell counts and plasma VL at the time of sample collection, are shown: “>log10 5.88” indicates VL was above the upper limit of detection, and was not quantified. Whether subjects received HU in addition to ART is indicated. Reverse transcriptase and p24 antigen EIA assay results, performed following virus isolation, are also shown: “ND” indicates culture supernatant was not tested using the RT assay; “NQ” indicates that the relevant result for the isolate was above or below the limit of detection for the assay and was not quantified; “−” indicates that virus isolation was attempted but RT activity or p24 antigen were not detected.
(0.32 MB DOC)

Table S2 Clinical and virus isolation data for PHAEDRA subjects. Shown are the clinical results and the results of attempted virus isolation from plasma obtained from PHAEDRA subjects. A single asterisk indicates the plasma sample from which virus isolation was attempted. Indicated by the column headings are the subject identification code, the phase of the PHAEDRA study at which the relevant sample was collected, and seroconversion status according to Fiebig et al [24]. Coincident CD4+ T cell counts and plasma VL at the time of sample collection are shown: “>log10 5.88” indicates VL was above the upper limit of detection, and was not quantified. Reverse transcriptase and p24 antigen EIA assay results, performed following virus isolation, are also shown: “ND” indicates culture supernatant was not tested using the RT assay; “NQ” indicates that the relevant result for the isolate was above or below the limit of detection for the assay and was not quantified; “−” indicates that virus isolation was attempted but RT activity or production of p24 antigen was not detected subsequently.
(0.17 MB DOC)

Table S3 Intra-assay variation analysis for the QPCR assay. To examine intra-assay variation, 20 replicates of each HIV-1 (A) and albumin (B) DNA standard were tested in the same run. Data represent the mean Ct value (Mean), standard deviation (SD) and coefficient of variation (COV, expressed as a percentage) for each standard. “N” indicates the number of replicates detected for each standard.
(0.04 MB DOC)

Table S4 Inter-assay variation analysis. To examine inter-assay variation, five consecutive runs with the HIV-1 (A) and albumin (B) DNA standards were performed. Standards were tested in triplicate within each run. Shown are the mean Ct values obtained for each standard following each of the five independent runs. Data represent the total number of replicates detected (N), mean Ct value (Mean), standard deviation (SD) and coefficient of variation (COV, expressed as a percentage) for each standard. Mean, SD and COV values were calculated using Ct values obtained for each replicate detected of the specified standard. A HIV-1 negative non-amplification control (NAC) was included, consisting of cellular DNA. ND indicates that the specified sample was not detected.
(0.06 MB DOC)
