# Supplementary material for: High Viral Fitness during Acute HIV-1 Infection
Source: PLoS One. 2010 Sep 9;5(9):e12631. doi: 10.1371/journal.pone.0012631 (PMC2936565; doi:10.1371/journal.pone.0012631)
Supplement: Text S1 — Detail of viral fitness QPCR assay validation. (0.04 MB DOC) [file pone.0012631.s001.doc]

**Text S1: Detection of total HIV-1 DNA production: QPCR assay validation**

A QPCR assay was developed and validated for detection of total HIV-1 DNA (extrachromosomal, integrated and 2LTR circular forms) production *ex vivo*. Parallel infection experiments were analysed using an HIV-1 DNA QPCR assay, developed and validated using two sets of primers and TaqMan probes targeting the HIV-1 LTR and the human albumin gene (see Table 1). The HIV-1 primers and probe were as previously used by Yun et al., (2002) and targeted a highly conserved region within the HIV-1 LTR [1,2]. This region was selected as the assay target following investigation of other conserved regions (*gag, env* and *pol*) within the HIV-1 genome (data not shown). Although the QPCR assay was designed to enable investigation of the relative replicative fitness of primary Australian HIV-1 isolates, predominantly subtype B [3], the HIV-1 primers and probe used enabled detection of a broad range of genetically diverse isolates. Using the QPCR assay, Group M subtype A (n=2), C (n=1), D (n=3) and F (n=2) virus strains were also detected (data not shown).

Albumin DNA was amplified in parallel with HIV-1 DNA for all samples tested. Amplification of albumin DNA enabled copies of HIV-1 DNA per cell to be quantified, and the efficiency of DNA extraction to be monitored. The primers and probes used in this study were those described previously [4].

**Assay sensitivity**

HIV-1 and albumin DNA standards were included in all real time PCR reactions to enable DNA quantification and to monitor reaction performance and efficiency. Human albumin DNA standards were generated by preparing 5 fold dilutions of cellular DNA extracted from donor PBMCs, with the standards ranging in concentration from 640 to 2 000 000 copies albumin DNA/5 µl. HIV-1 DNA standards were prepared by diluting a known concentration of HIV-1_IIIB_ DNA (Advanced Biotechnologies Inc., USA) in DNA extracted from donor PBMCs, at a concentration of 200 000 cell equivalents/100 µl. The HIV-1 DNA standards were diluted in donor human DNA to stabilise the viral DNA during storage and to best mimic conditions of test samples. The standard range was 2 to 6 000 copies HIV-1_IIIB_ DNA/5 μl. The range of both the albumin and HIV-1 standard curves was selected to ensure that DNA copies calculated for test samples were not derived by extrapolation.

Using the quantified DNA standards, the limit of analytical sensitivity was estimated for both the HIV-1 and albumin QPCR reactions. The lower limit was defined as the standard containing the lowest concentration of HIV-1 or albumin DNA that was detected in 100% of reactions. The lower limit of sensitivity was estimated to be 10 and 640 DNA copies for the HIV-1 and albumin QPCR reactions, respectively (Supplementary Table 3).

**Intra and inter-assay variation**

To investigate intra-assay reproducibility, 20 replicates of each quantified albumin and HIV-1 standard were amplified within the same run to enable calculation of a coefficient of variation (expressed as a percentage, %COV) for each standard (Supplementary Table 3). A COV of <5% indicated minimal variation between replicates. The %COV calculated for each albumin DNA standard were <1.5%, and <2.2% for each HIV-1 DNA standard. A mean threshold cycle (Ct) and standard deviation (SD) value was also calculated for each standard. These were used as reference values to further monitor the amplification efficiency of all subsequent QPCR reactions.

Inter-assay reproducibility was examined by performing five consecutive runs with the HIV-1 and albumin DNA standards in triplicate. To ensure Ct values were within the acceptable range, Ct values obtained following inter-assay variation testing were compared with the mean and SD values previously calculated for each standard following intra-assay variation testing. The COV calculated for each albumin DNA standard was <4.3%, and <3.2% for each HIV-1 DNA standard (Supplementary Table 4). The minimal inter- and intra- assay variation demonstrated the high reproducibility of the QPCR assay.

**References**

1. Yun Z, Fredriksson E, Sonnerborg A (2002) Quantification of human immunodeficiency virus type 1 proviral DNA by the TaqMan real-time PCR assay. J Clin Microbiol 40: 3883-3884.

2. Avettand-Fenoel V, Chaix ML, Blanche S, Burgard M, Floch C, et al. (2009) LTR real-time PCR for HIV-1 DNA quantitation in blood cells for early diagnosis in infants born to seropositive mothers treated in HAART area (ANRS CO 01). J Med Virol 81: 217-223.

3. Hemelaar J, Gouws E, Ghys PD, Osmanov S (2006) Global and regional distribution of HIV-1 genetic subtypes and recombinants in 2004. Aids 20: W13-23.

4. Desire N, Dehee A, Schneider V, Jacomet C, Goujon C, et al. (2001) Quantification of human immunodeficiency virus type 1 proviral load by a TaqMan real-time PCR assay. J Clin Microbiol 39: 1303-1310.
