# Supplementary material for: High Viral Fitness during Acute HIV-1 Infection
Source: PLoS One. 2010 Sep 9;5(9):e12631. doi: 10.1371/journal.pone.0012631 (PMC2936565; doi:10.1371/journal.pone.0012631)
Supplement: Table S1 — Clinical and virus isolation data for PULSE subjects. Shown are the clinical results and the results of attempted virus isolation from plasma or PBMCs obtained from PULSE subjects; “triangle” indicates that virus isolation was attempted from the sample indicated. A single asterisk indicates the sample used for virus isolation was plasma; a double asterisk indicates that virus isolation was attempted from PBMCs when either plasma was not available or virus isolation from plasma was unsuccessful. Shown is the subject identification number followed by the phase of the PULSE study during which the sample was collected. “A”, “B” and “C” indicate PULSE study Phases A, B and C. The subsequent number indicates during which of up to three B or C phases sample collection occurred; prefaced by “W” (weeks), the following number indicates duration of the specified phase at sample collection. Seroconversion status according to the Fiebig et al [24] stages, coincident CD4+ T cell counts and plasma VL at the time of sample collection, are shown: “>log10 5.88” indicates VL was above the upper limit of detection, and was not quantified. Whether subjects received HU in addition to ART is indicated. Reverse transcriptase and p24 antigen EIA assay results, performed following virus isolation, are also shown: “ND” indicates culture supernatant was not tested using the RT assay; “NQ” indicates that the relevant result for the isolate was above or below the limit of detection for the assay and was not quantified; “−” indicates that virus isolation was attempted but RT activity or p24 antigen were not detected. (0.32 MB DOC) [file pone.0012631.s002.doc]

| **Subject**  **Table S1: Clinical and virus isolation data for PULSE subjects**  **identifier** | **Phase** | **Seroconversion**  **status at baseline (ref 24)** | **CD4+ T cells (cells/µl)** | **HU** | **Viral load (log_10_ RNA copies/ml)** | **RT activity in viral stock (cpm/µl)** | **p24 antigen (pg/ml)** |
| --- | --- | --- | --- | --- | --- | --- | --- |
| **1.01** | Baseline^Δ^ | Stage VI | 864 | **+** | 4.52 | - | - |
|  | B1 W2 |  | 920 |  | <1.70 |  |  |
|  | B1 W24 |  | 846 |  | 3.25 |  |  |
|  | Phase D |  | 720 |  | 3.14 |  |  |
| **1.03** | Baseline^Δ^* | Stage VI | 468 | **-** | 6.29 | 5 715 | 1 894 882 |
|  | A W24 |  | 814 |  | 1.90 |  |  |
|  | A W52 |  | 950 |  | <1.70 |  |  |
|  | B1 W2^Δ^* |  | 640 |  | 5.76 | ND | 375 000 |
|  | C1W2 |  | 875 |  | 3.64 |  |  |
|  | C1 W12 |  | 782 |  | 2.23 |  |  |
|  | B2 W2 |  | 575 |  | 4.27 |  |  |
|  | C2 W0^Δ^** |  | NA |  | 5.88 | ND | NQ |
|  | C2 W2 |  | 780 |  | 3.73 |  |  |
|  | C2 W12 |  | 1131 |  | 2.40 |  |  |
|  | B3 W2^Δ^** |  | NA |  | 5.00 | - | - |
|  | PhD^Δ^* |  | 870 |  | 5.50 | ND | 417 000 |
| **1.04** | Baseline^Δ^* | Stage VI | 310 | **+** | 5.24 | 711 | 25 210 |
|  | A W24 |  | 490 |  | <1.70 |  |  |
|  | A W52 |  | 315 |  | <1.70 |  |  |
|  | B1 W2 |  | 297 |  | 3.63 |  |  |
|  | B1 W3^Δ^* |  | 330 |  | 4.46 | - | - |
|  | C1 W0^Δ^** |  | NA |  | 4.79 | - | - |
|  | C1 W2 |  | 460 |  | 2.60 |  |  |
|  | C1 W12 |  | 336 |  | <1.70 |  |  |
|  | B2 W2^Δ^* |  | 324 |  | 3.56 | - | - |
|  | C2 W2 |  | 442 |  | 2.69 |  |  |
|  | C2 W12 |  | 504 |  | 2.60 |  |  |
|  | B3 W4^Δ^** |  | 399 |  | 4.12 | - | - |
| **2.05** | Baseline^Δ^* | Stage V | 270 | **-** | 5.68 | - | - |
|  | B1 W3 |  | 756 |  | NA |  |  |
|  | B1 W24 |  | 768 |  | 2.64 |  |  |
|  | Phase D |  | 792 |  | 4.04 |  |  |
| **3.04** | Baseline^Δ^* | Stage V | 693 | **-** | 4.81 | - | - |
|  | A W24 |  | 836 |  | <1.70 |  |  |
|  | A W52 |  | 945 |  | <1.70 |  |  |
|  | B1 W2 |  | 850 |  | 4.27 |  |  |
|  | C1 W0^Δ^** |  | NA |  | 4.88 | - | - |
|  | C1 W2 |  | 420 |  | <1.70 |  |  |
|  | C1 W12 |  | 792 |  | <1.70 |  |  |
|  | B2 W2 |  | 544 |  | 3.08 |  |  |
|  | B2 W4 |  | 646 |  | 4.12 |  |  |
|  | C2 W12 |  | 1 085 |  | <1.70 |  |  |
|  | B3 W4 |  | 960 |  | 1.90 |  |  |
|  | B3 W24^Δ^** |  | 792 |  | 2.56 | ND | 15 |
| **3.06** | Baseline^Δ^ | Stage IV | 539 | **-** | 6.31 | 55 | 1 666 |
|  | A W52 |  | 957 |  | <1.70 |  |  |
|  | B1 W2 |  | 740 |  | 3.50 |  |  |
|  | B1 W3^Δ^* |  | 1 066 |  | 4.30 | - | - |
|  | C1 W0^Δ^** |  | NA |  | 5.21 | ND | NQ |
|  | C1 W12 |  | 744 |  | <1.70 |  |  |
|  | B2 W2 |  | 1 020 |  | 2.84 |  |  |
|  | B2 W4^Δ^* |  | 1 044 |  | 4.05 | - | - |
|  | C2 W2 |  | 1 008 |  | 2.60 |  |  |
|  | C2 W12 |  | 930 |  | <1.70 |  |  |
|  | B3 W4 |  | 900 |  | 3.48 |  |  |
|  | PhD^Δ^* |  | 864 |  | 2.76 | - | - |
| **3.07** | Baseline^Δ^* | Stage IV | 629 | **+** | 5.89 | 146 | 7 344 |
|  | A W24 |  | 782 |  | 2.08 |  |  |
|  | A W52 |  | 768 |  | <1.70 |  |  |
|  | B1 W2 |  | 612 |  | 3.52 |  |  |
|  | B1 W3^Δ^* |  | 576 |  | 4.13 | - | - |
|  | C1 W2 |  | 672 |  | 2.85 |  |  |
|  | C1 W12 |  | 759 |  | <1.70 |  |  |
|  | B2 W2^Δ^* |  | 714 |  | 3.23 | - | - |
|  | B2 W4 |  | 736 |  | 3.30 |  |  |
|  | C2 W2 |  | 690 |  | 2.53 |  |  |
|  | B3 W4 |  | 693 |  | 3.74 |  |  |
|  | PhD^Δ^* |  | 544 |  | ND | ND | NQ |
| **3.10** | Baseline* | Stage VI | 374 |  | 5.18 | 17 | NQ |
|  | A W24 |  | 528 |  | 1.85 |  |  |
|  | A W52 |  | 528 |  | <1.70 |  |  |
|  | B1 W2 |  | 400 |  | 3.54 |  |  |
|  | B1 W3 ^Δ^* |  | 324 |  | 4.62 | - | - |
|  | C1 W2 |  | 504 |  | 2.90 |  |  |
|  | C1 W12 |  | 475 | **+** | 2.15 |  |  |
|  | B2 W2 |  | 512 |  | 1.85 |  |  |
|  | B2 W4 ^Δ^* |  | 600 |  | 3.73 | - | - |
|  | C2 W0 |  | NA |  | 4.33 |  |  |
|  | C2 W2 ^Δ^** |  | 570 |  | 2.15 | - | - |
|  | C2 W12 |  | 456 |  | <1.70 |  |  |
|  | B3 W4 ^Δ^** |  | 621 |  | 4.83 | - | - |
| **3.12** | Baseline^Δ^* | Stage IV | 833 | **-** | ≥5.88 | 2 008 | 71 180 |
|  | A W24 |  | 1 302 |  | <1.70 |  |  |
|  | A W52 |  | 945 |  | <1.70 |  |  |
|  | B1 W2 |  | 1 092 |  | 2.08 |  |  |
|  | B1 W3 |  | 629 |  | 3.64 |  |  |
|  | B1 W4 ^Δ^* |  | 840 |  | 4.67 | - | - |
|  | C1 W2 |  | NA |  | 2.23 |  |  |
|  | C1 W12 |  | 1 008 |  | <1.70 |  |  |
|  | B2 W4 |  | 740 |  | 3.87 |  |  |
|  | C2 W2 |  | 884 |  | 2.20 |  |  |
|  | C2 W12 |  | 861 |  | <1.70 |  |  |
|  | B3 W4 |  | 656 |  | 3.64 |  |  |
| **3.13** | Baseline^Δ^* | Stage V | 390 | **-** | 5.99 | 2 586 | 335 813 |
|  | A W24 |  | 510 |  | <1.70 |  |  |
|  | A W52 |  | 640 |  | <1.70 |  |  |
|  | B1 W2 |  | 544 |  | <1.70 |  |  |
|  | B1 W3 |  | 480 |  | 2.40 |  |  |
|  | B1 W4 |  | 576 |  | 2.64 |  |  |
|  | B1 W24 |  | 510 |  | 3.11 |  |  |
|  | PhD |  | 429 |  | 3.23 |  |  |
| **3.19** | Baseline^Δ^* | Stage IV | 972 | **-** | 5.82 | 1 084 | 36 920 |
|  | A W24 |  | 1 148 |  | <1.70 |  |  |
|  | B1 W2 |  | 1 218 |  | 2.64 |  |  |
|  | B1 W3 |  | 1 196 |  | 4.00 |  |  |
|  | C1 W12 |  | 1 628 |  | <1.70 |  |  |
|  | B2 W4 |  | NA |  | 3.68 |  |  |
| **3.21** | Baseline^Δ^* | Stage IV | 266 | **+** | 6.88 | 50 | 598 |
|  | A W24 |  | 608 |  | <1.70 |  |  |
|  | B1 W2 |  | 296 |  | <1.70 |  |  |
|  | B1 W4 ^Δ^* |  | 352 |  | 4.72 | - | - |
|  | C1 W0^Δ^** |  | NA |  | 4.80 | ND | NQ |
|  | C1 W2 |  | 405 |  | 2.23 |  |  |
|  | C1 W12 |  | NA |  | <1.70 |  |  |
|  | B2 W2 |  | NA |  | 2.18 |  |  |
|  | B2 W4 |  | NA |  | 4.32 |  |  |
|  | B3 W4 ^Δ^** |  | NA |  | 3.26 | - | - |
| **4.02** | Baseline ^Δ^* | Stage V | 437 | **+** | 6.03 | 683 | 24 345 |
|  | A W24 |  | 592 |  | 1.78 |  |  |
|  | A W52 |  | 561 |  | <1.70 |  |  |
|  | B1 W2 ^Δ^* |  | 612 |  | 5.66 | NQ | 146 |
|  | C1 W2 |  | 598 |  | 3.32 |  |  |
|  | C1 W12 |  | 494 |  | 1.90 |  |  |
|  | B2 W2 |  | NA |  | 3.88 |  |  |
|  | C2 W2 |  | 480 |  | 2.60 |  |  |
|  | C2 W12 |  | 429 |  | <1.70 |  |  |
|  | B3 W4 ^Δ^ ** |  | 504 |  | 4.28 | ND | 509 000 |
| **4.03** | Baseline ^Δ^ * | Stage IV | 638 | **+** | 6.79 | 844 | 86 009 |
|  | A W24 |  | 561 |  | <1.70 |  |  |
|  | A W52 |  | 928 |  | <1.70 |  |  |
|  | B1 W2 |  | 714 |  | <1.70 |  |  |
|  | B1 W3 |  | 704 |  | 2.15 |  |  |
|  | B1 W24 |  | 665 |  | 3.70 |  |  |
|  | C1 W2 |  | 792 |  | <1.70 |  |  |
|  | C1 W12 |  | 646 |  | <1.70 |  |  |
|  | B2 W2 |  | 504 |  | <1.70 |  |  |
|  | B2 W4 |  | 435 |  | 2.49 |  |  |
|  | B2 W12 |  | 650 |  | 3.96 |  |  |
| **4.05** | Baseline ^Δ^ * | Stage V | 720 | **+** | 5.36 | - | - |
|  | B1 W2 |  | 384 |  | 3.48 |  |  |
|  | B1 W3 |  | 481 |  | 3.87 |  |  |
| **4.06** | Baseline ^Δ^ * | Stage IV | 567 | **-** | 6.55 | 4 380 | 569 962 |
|  | A W24 |  | 902 |  | <1.70 |  |  |
|  | A W52 |  | 1 000 |  | <1.70 |  |  |
|  | B1 W2 |  | 924 |  | 2.41 |  |  |
|  | B1 W3 |  | 798 |  | 3.61 |  |  |
|  | B1 W4 ^Δ^ * |  | 780 |  | 3.76 | ND | NQ |
|  | B3 W16 ^Δ^ ** |  | NA |  | 4.03 | - | - |
| **4.07** | Baseline ^Δ^ * | Stage IV | 480 | **-** | 6.08 | 26 | 167 |
|  | C1 W0 ^Δ^ ** |  | NA |  | 5.23 | ND | UQ |
|  | B2 W2 ^Δ^ * |  | NA |  | 4.64 | - | - |
| **4.10** | Baseline ^Δ^ * | Stage IV | 540 | **-** | 6.29 | 1 323 | 33 512 |
|  | A W24 |  | 1 200 |  | <1.70 |  |  |
|  | B1 W2 |  | 703 |  | 2.15 |  |  |
|  | B1 W3 |  | 1 026 |  | 3.87 |  |  |
| **5.01** | Baseline ^Δ^ * | Stage IV | 420 | **-** | 5.20 | 821 | 24 735 |
|  | B1 W2 |  | 600 |  | <1.70 |  |  |
|  | B1 W24 |  | 476 |  | 3.70 |  |  |
|  | Phase D ^Δ^ ** |  | 640 |  | 2.42 | ND | NQ |
| **5.03** | Baseline ^Δ^ * | Stage VI | 260 | **+** | 4.14 | - | - |
|  | B1 W2 |  | 595 |  | 3.04 |  |  |
|  | B1 W4 |  | 400 |  | 2.83 |  |  |
