# Supplementary material for: High Viral Fitness during Acute HIV-1 Infection
Source: PLoS One. 2010 Sep 9;5(9):e12631. doi: 10.1371/journal.pone.0012631 (PMC2936565; doi:10.1371/journal.pone.0012631)
Supplement: Table S2 — Clinical and virus isolation data for PHAEDRA subjects. Shown are the clinical results and the results of attempted virus isolation from plasma obtained from PHAEDRA subjects. A single asterisk indicates the plasma sample from which virus isolation was attempted. Indicated by the column headings are the subject identification code, the phase of the PHAEDRA study at which the relevant sample was collected, and seroconversion status according to Fiebig et al [24]. Coincident CD4+ T cell counts and plasma VL at the time of sample collection are shown: “>log10 5.88” indicates VL was above the upper limit of detection, and was not quantified. Reverse transcriptase and p24 antigen EIA assay results, performed following virus isolation, are also shown: “ND” indicates culture supernatant was not tested using the RT assay; “NQ” indicates that the relevant result for the isolate was above or below the limit of detection for the assay and was not quantified; “−” indicates that virus isolation was attempted but RT activity or production of p24 antigen was not detected subsequently. (0.17 MB DOC) [file pone.0012631.s003.doc]

Table 2: Viral fitness scores and clinical data for acute HIV-1 infecti

| **Subject identifier**  **Table S2: Clinical and virus isolation data for PHAEDRA subjects** | **Weeks post baseline** | **Seroconversion status at baseline**  **(ref 24)** | **CD4+ T cells (cells/µl)** | **Viral load (RNA copies/ml)** | **RT activity in viral stock (cpm/µl)** | **p24 antigen (pg/ml)** |
| --- | --- | --- | --- | --- | --- | --- |
| **PSO1009** | Baseline* | Stage VI | 774 | 5.38 | 130 | 110 000 |
|  | 4 |  | 513 | 5.39 |  |  |
|  | 8 |  | 576 | 5.09 |  |  |
|  | 36 |  | 521 | 5.34 |  |  |
|  | 52* |  | 285 | 5.88 | 4 666 | 38 900 |
| **PSO1014** | Baseline* | Stage VI | 619 | 4.33 | 691 | 3 210 |
|  | 1 |  | 597 | 4.23 |  |  |
|  | 8 |  | 636 | 5.15 |  |  |
|  | 24 |  | 552 | 4.27 |  |  |
|  | 36 |  | 597 | 3.72 |  |  |
|  | 52* |  | 390 | 3.97 | 2 794 | 353 000 |
| **PSO1019** | Baseline | Stage VI | 1 266 | 2.60 | - | - |
|  | 4 |  | 1 433 | 3.24 |  |  |
|  | 8 |  | 1 220 | 2.74 |  |  |
|  | 24 |  | 1 213 | 2.89 | - | - |
| **PSO1020** | Baseline | Stage VI | 847 | 5.31 | 125 | 16 |
|  | 1 |  | 639 | 5.43 |  |  |
|  | 4 |  | 758 | 5.88 |  |  |
|  | 8 |  | 735 | 4.92 |  |  |
|  | 24 |  | 756 | 5.45 |  |  |
|  | 36 |  | 753 | 4.71 | - | - |
| **PSO1024** | Baseline* | Stage VI | 537 | 5.40 | 3 006 | 397 000 |
|  | 1 |  | 643 | 5.88 |  |  |
|  | 4 |  | 608 | 5.40 |  |  |
|  | 24 |  | 662 | 5.20 |  |  |
|  | 36* |  | 633 | 4.95 | 2 927 | NQ |
| **PSO1026** | Baseline* | Stage VI | 1 266 | 4.58 | 3 335 | NQ |
|  | 4 |  | NA | 4.84 |  |  |
|  | 24 |  | 728 | 4.10 |  |  |
|  | 36* |  | 792 | 4.52 | 1 646 | 257 000 |
| **PSO1027** | Baseline | Stage VI | 1 196 | 3.95 | - | - |
|  | 8 |  | 1 075 | 4.01 |  |  |
|  | 36 |  | 711 | 3.79 | - | - |
| **PSO1029** | Baseline | Stage VI | 313 | 4.18 | 145 | ND |
|  | 1 |  | NA | ND |  |  |
|  | 24 |  | 364 | 4.30 |  |  |
|  | 36 |  | 321 | 4.79 | 3 687 | 257 000 |
| **PSO2010** | Baseline* | Stage VI | 644 | 5.75 | 2 468 | 665 000 |
|  | 52* |  | 456 | 5.18 | 3 925 | 138 000 |
| **PSO2011** | Baseline | Stage VI | 335 | 5.02 | 2 082 | 212 000 |
|  | 1 |  | 475 | 5.58 |  |  |
|  | 4 |  | 460 | 5.71 |  |  |
|  | 52 |  | 252 | 5.42 | NQ | 271 |
| **PSO2016** | Baseline* | Stage V | 380 | 5.88 | 889 | 976 000 |
|  | 1 |  | 726 | 3.94 |  |  |
|  | 4 |  | NA | NA |  |  |
|  | 8 |  | 504 | 4.63 |  |  |
|  | 24 |  | 576 | 4.51 |  |  |
|  | 36* |  | NA | 4.25 | 1 107 | 935 000 |
| **PSO2019** | Baseline* | Stage V | 630 | 25 700 | 257 | 3 |
|  | 1 |  | 660 | 15 800 |  |  |
|  | 4 |  | 646 | 11 600 |  |  |
|  | 8 |  | 700 | 80 700 |  |  |
|  | 24 |  | 522 | 60 100 |  |  |
|  | 36* |  | 440 | 100 000 | 2 182 | 609 000 |
| **PSO4017** | Baseline* | Stage VI | 832 | 77 400 | ND | 8 |
|  | 1 |  | 775 | 64 200 |  |  |
|  | 4 |  | 840 | 64 300 |  |  |
|  | 8 |  | 756 | 58 500 |  |  |
|  | 36 |  | 621 | 42 900 |  |  |
|  | 52* |  | 600 | 52 900 | ND | 28 |
| **PSO4028** | Baseline | Stage VI | 589 | 71 600 | 86.5 | 51 900 |
|  | 1 |  | 406 | 147 000 |  |  |
|  | 4 |  | 416 | 150 000 |  |  |
|  | 24 |  | NA | 128 000 |  |  |
|  | 36 |  | 289 | ND | 298 | 379 000 |
| **PSO4029** | Baseline* | Stage VI | 680 | 218 000 | ND | 1 660 |
|  | 1 |  | 693 | 181 000 |  |  |
|  | 4 |  | 627 | 29 500 |  |  |
|  | 8 |  | 684 | 27 600 |  |  |
|  | 24 |  | 520 | 21 000 |  |  |
|  | 36* |  | 493 | 27 000 | ND | 28 |
| **PSO4032** | Baseline | Stage VI | 588 | 52 300 | - | - |
|  | 1 |  | 420 | 63 300 |  |  |
|  | 4 |  | 464 | 54 800 |  |  |
|  | 8 |  | 390 | 81 400 |  |  |
|  | 24 |  | 299 | 285 000 |  |  |
|  | 36 |  | 360 | 72 500 | - | - |
| **PSO4036** | Baseline | Stage VI | 756 | 66 600 | - | - |
|  | 1 |  | 522 | 750 000 |  |  |
|  | 4 |  | 676 | 10 200 |  |  |
|  | 8 |  | 552 | 20 400 |  |  |
|  | 24 |  | 546 | 51 100 |  |  |
|  | 36 |  | 648 | 10 100 | - | - |
