# Supplementary material for: High Viral Fitness during Acute HIV-1 Infection
Source: PLoS One. 2010 Sep 9;5(9):e12631. doi: 10.1371/journal.pone.0012631 (PMC2936565; doi:10.1371/journal.pone.0012631)
Supplement: Table S3 — Intra-assay variation analysis for the QPCR assay. To examine intra-assay variation, 20 replicates of each HIV-1 (A) and albumin (B) DNA standard were tested in the same run. Data represent the mean Ct value (Mean), standard deviation (SD) and coefficient of variation (COV, expressed as a percentage) for each standard. “N” indicates the number of replicates detected for each standard. (0.04 MB DOC) [file pone.0012631.s004.doc]

**Table S3: Intra-assay variation analysis for the QPCR assay**

**A**

|  | **Copies of HIV-1 DNA/reaction** | | | | | |
| --- | --- | --- | --- | --- | --- | --- |
|  | **6 000** | **1 200** | **240** | **48** | **10** | **2** |
| **N** | 20.0 | 20.0 | 20.0 | 20.0 | 19.0 | 10.0 |
| **Mean** | 25.2 | 27.6 | 30.0 | 33.5 | 36.7 | 40.3 |
| **SD** | 0.6 | 0.3 | 0.5 | 0.4 | 0.8 | 0.9 |
| **Range (+/- 2 SD)** | 1.2 | 0.6 | 1.0 | 0.8 | 1.6 | 1.8 |
| **COV (%)** | 2.3 | 1.0 | 1.5 | 1.2 | 2.0 | 2.2 |
|  |  | | | | | |
|  | **Copies of Albumin DNA/reaction** | | | | | |
|  | **2 000 000** | **400 000** | **80 000** | **16 000** | **3 200** | **640** |
| **N** | 20.0 | 18.0 | 20.0 | 20.0 | 20.0 | 20.0 |
| **Mean** | 20.5 | 21.6 | 24.4 | 26.7 | 28.9 | 31.4 |
| **SD** | 0.2 | 0.1 | 0.1 | 0.2 | 0.3 | 0.5 |
| **Range (+/- 2 SD)** | 0.4 | 0.2 | 0.2 | 0.4 | 0.6 | 1.0 |
| **COV (%)** | 1.0 | 0.3 | 0.5 | 0.8 | 0.9 | 1.4 |

**B**
