# Supplementary material for: High Viral Fitness during Acute HIV-1 Infection
Source: PLoS One. 2010 Sep 9;5(9):e12631. doi: 10.1371/journal.pone.0012631 (PMC2936565; doi:10.1371/journal.pone.0012631)
Supplement: Table S4 — Inter-assay variation analysis. To examine inter-assay variation, five consecutive runs with the HIV-1 (A) and albumin (B) DNA standards were performed. Standards were tested in triplicate within each run. Shown are the mean Ct values obtained for each standard following each of the five independent runs. Data represent the total number of replicates detected (N), mean Ct value (Mean), standard deviation (SD) and coefficient of variation (COV, expressed as a percentage) for each standard. Mean, SD and COV values were calculated using Ct values obtained for each replicate detected of the specified standard. A HIV-1 negative non-amplification control (NAC) was included, consisting of cellular DNA. ND indicates that the specified sample was not detected. (0.06 MB DOC) [file pone.0012631.s005.doc]

**Table S4: Inter-assay variation analysis**

**A**

|  | **Copies of HIV-1 DNA** | | | | | | |
| --- | --- | --- | --- | --- | --- | --- | --- |
|  | **6 000** | **1 200** | **240** | **48** | **10** | **2** | **NAC** |
| **1** | 28.0 | 28.0 | 31.0 | 33.0 | 36.0 | ND | ND |
| **2** | 29.0 | 29.0 | 31.0 | 33.0 | 36.0 | ND | ND |
| **3** | 28.0 | 28.0 | 31.0 | 33.0 | 36.0 | ND | ND |
| **4** | 27.0 | 27.0 | 29.0 | 33.0 | 35.0 | ND | ND |
| **5** | 27.0 | 27.0 | 30.0 | 32.0 | 34.0 | ND | ND |
| **N** | 15.0 | 15.0 | 15.0 | 15.0 | 15.0 | 15.0 | 15.0 |
| **Mean** | 28.3 | 28.3 | 30.8 | 33.1 | 35.9 | 0 | 0 |
| **SD** | 0.9 | 0.9 | 0.6 | 0.7 | 0.9 | 0 | 0 |
| **COV (%)** | 3.2 | 3.2 | 2.1 | 2.0 | 2.5 | 0 | 0 |

**B**

|  | | **Copies of Albumin DNA** | | | | | | |
| --- | --- | --- | --- | --- | --- | --- | --- | --- |
|  | | **2 000 000** | **400 000** | **80 000** | **16 000** | **3 200** | **640** | **NAC** |
| **1** | 23.6 | | 26.4 | 28.8 | 30.5 | 33.4 | 35.9 | ND |
| **2** | 23.5 | | 25.3 | 27.8 | 32.7 | 34.8 | ND | ND |
| **3** | 23.0 | | 25.2 | 27.6 | 30.1 | 32.8 | 35.8 | ND |
| **4** | 23.3 | | 25.5 | 27.9 | 30.2 | 33.1 | 35.8 | ND |
| **5** | 24.0 | | 25.8 | 28.3 | 32.9 | 35.1 | 36.2 | ND |
| **N** | 15.0 | | 15.0 | 15.0 | 15.0 | 15.0 | 15.0 | 15.0 |
| **Mean** | 23.5 | | 25.6 | 28.1 | 31.3 | 33.8 | 35.9 | 0 |
| **SD** | 0.4 | | 0.5 | 0.4 | 1.4 | 1.0 | 0.2 | 0 |
| **COV (%)** | 1.5 | | 1.9 | 1.6 | 4.4 | 3.0 | 0.5 | 0 |
